# Case Report: Upadacitinib and apremilast combination therapy for guselkumab-induced paradoxical eczema in psoriasis

**DOI:** 10.3389/fimmu.2026.1696255

**Published:** 2026-04-17

**Authors:** Luan Yang, Jing Zhou, Zhenqiang Ruan, Keshi Shi

**Affiliations:** Department of Dermatology, Jinan Dermatosis Prevention and Control Hospital, Jinan, Shandong, China

**Keywords:** apremilast, deucravacitinib, paradoxical reaction, psoriasis, upadacitinib

## Abstract

Psoriasis is an immune-mediated inflammatory skin disease. Although biologics have markedly improved psoriasis symptoms, they may occasionally trigger paradoxical inflammatory reactions, particularly with IL-17A or TNF-α inhibitors. However, paradoxical inflammatory reactions associated with the IL-23 inhibitor guselkumab is exceptionally rare. We describe the case of a 70-year-old male with a 5-year history of psoriasis who had previously received tofacitinib for one year without achieving complete clearance. Following the initiation of guselkumab, the patient developed paradoxical eczema. Subsequent treatment with cyclosporine and the TYK2 inhibitor deucravacitinib proved ineffective. Ultimately, combination therapy with the JAK1 inhibitor upadacitinib and the PDE4 inhibitor apremilast achieved satisfactory control of both the paradoxical eczema and the underlying psoriatic lesions. This case highlights that upadacitinib used in combination with apremilast can effectively reverse paradoxical inflammatory reactions while sustaining psoriasis control. This dual-targeting strategy offers a novel therapeutic option for biologic-induced paradoxical reactions, and its broad-spectrum anti-inflammatory properties and potential synergistic effects warrant further clinical investigation.

## 1 Introduction

Psoriasis is a multifactorial immune-mediated skin disorder influenced by genetic predisposition, environmental triggers, and immune dysregulation. Its pathogenesis is predominantly driven by T helper (Th)17- and Th1-mediated inflammatory pathways, with the IL-23/IL-17 axis playing a central role. The advent of biologic therapies has markedly improved disease control, particularly with inhibitors targeting TNF-α, IL-12/23, IL-17A, and IL-23p19. However, biologic agents may occasionally induce paradoxical inflammatory reactions—manifesting as the emergence of new inflammatory conditions or the exacerbation of pre-existing diseases. Reported clinical phenotypes include paradoxical psoriasis, arthritis, inflammatory bowel disease, hidradenitis suppurativa, uveitis, palmoplantar pustulosis, and eczema (1). Such events are most frequently reported with TNF-α or IL-17 inhibitors, while cases linked to IL-12/23p40 or IL-23p19 inhibitors are uncommon, particularly IL-23p19 inhibitors such as guselkumab, risankizumab and tildrakizumab are exceptionally rare (2). Management strategies typically involve discontinuing the offending biologic, switching to an alternative therapeutic target, or reverting to conventional treatments. Compared with biologics, oral small-molecule targeted agents provide advantages such as ease of administration and broader immunomodulatory activity. This report presents a case of guselkumab-induced paradoxical eczema successfully treated with the combination of upadacitinib and apremilast, highlighting a potential oral dual-target approach for reversing paradoxical inflammatory reactions while maintaining psoriasis control.

## 2 Case report

**Figure 1:**
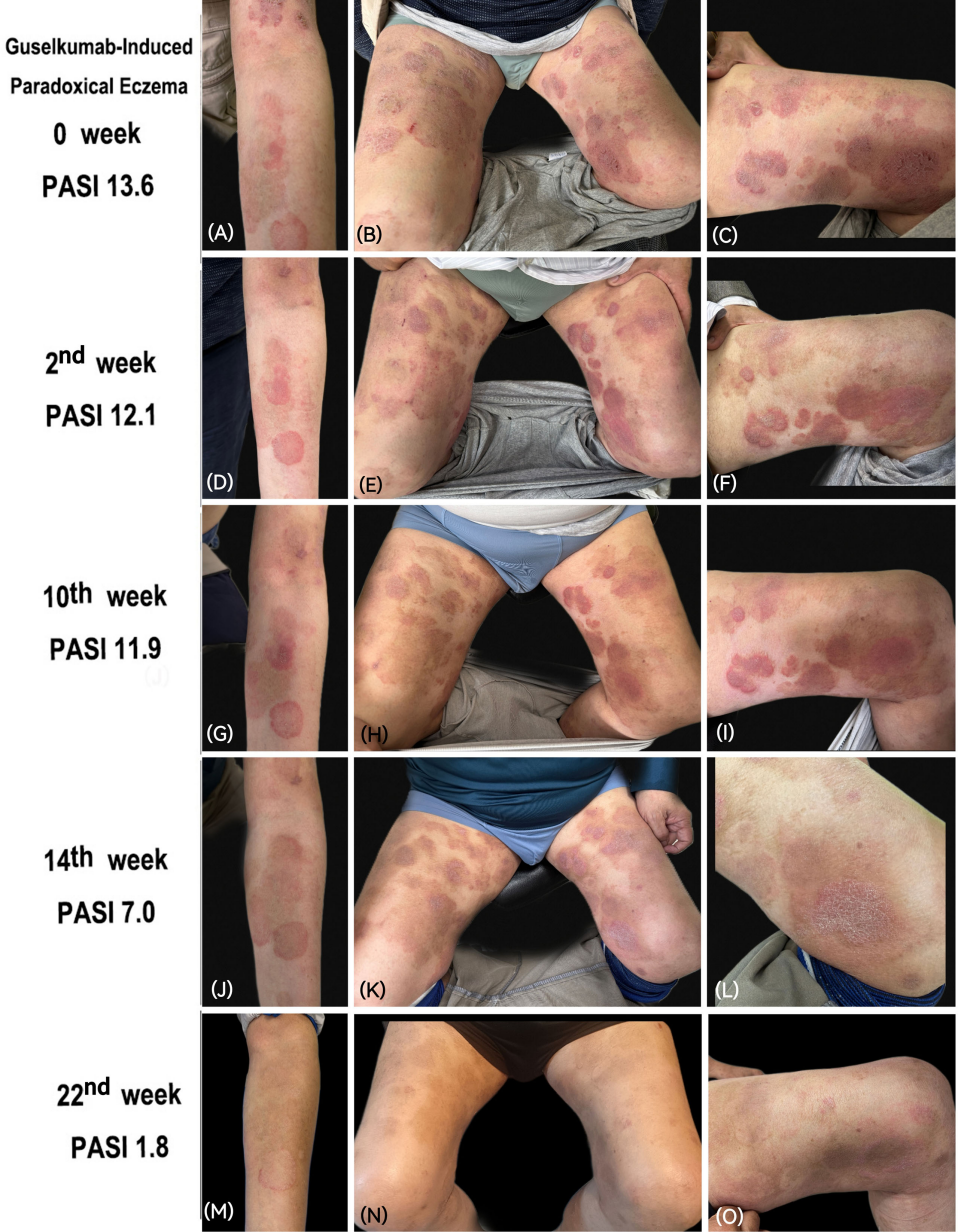
Clinical photographs. **(A–C)** Clinical presentation after 2 weeks of guselkumab treatment; **(D–F)** Presentation after 2 weeks of cyclosporine treatment; **(G–I)** Presentation after 8 weeks of deucravacitinib treatment; **(J–L)** Presentation after 4 weeks of combined tofacitinib and apremilast therapy; **(M–O)** Presentation after 8 weeks of combined upadacitinib and apremilast therapy.

A 70-year-old male presented with a 5-year history of progressive plaque psoriasis. The initial presentation was characterized by symmetrically distributed, scaly plaques exhibiting classic Auspitz sign and a fine membrane upon scale removal. The lesions predominantly involved the extremities, with minimal involvement of the scalp and trunk. The patient reported no arthralgia or symptoms suggestive of psoriatic arthritis, and there was no involvement of special sites. He denied any personal history of atopy, eczema, or other allergic conditions since childhood. The patient had a 20-year history of hypertension, which was well-controlled with a regimen of olmesartan hydrochlorothiazide and metoprolol sustained-release tablets. Prior to presentation at our institution, the patient had received various therapies including oral methotrexate, topical corticosteroids and vitamin D3 analogs, all with suboptimal efficacy. Cyclosporine and acitretin were not considered due to concerns regarding his advanced age and underlying hypertension. Over the past year, his psoriatic lesions worsened with pruritus. Consequently, treatment with tofacitinib was initiated, achieving partial control of skin lesions but failing to achieve a 75% improvement in the psoriasis area and severity index (PASI). Following exclusion of contraindications for biologics, guselkumab was initiated (100 mg, single injection). Three days post-injection, the patient developed marked pruritus. By the second week, both pruritus and lesions worsened progressively, with existing erythematous plaques becoming more intense, raised, and expanded in size, accompanied by newly developed discoid erythema. The pruritus was severe (visual analogue scale: 8/10), significantly impairing his sleep quality. He denied any other chronic illnesses or family history of similar disorders. Systemic examination revealed no abnormalities. Dermatological examination demonstrated symmetrical erythematous plaques with overlying silvery-white scales on the trunk and extremities, some of which coalesced into larger patches. Additionally, round and patchy edematous erythema were observed on the limbs, PASI score 13.6 (**Figures 1A–C**). Written informed consent was obtained from the patient for publication of this case report and any accompanying images.

**Figure 2:**
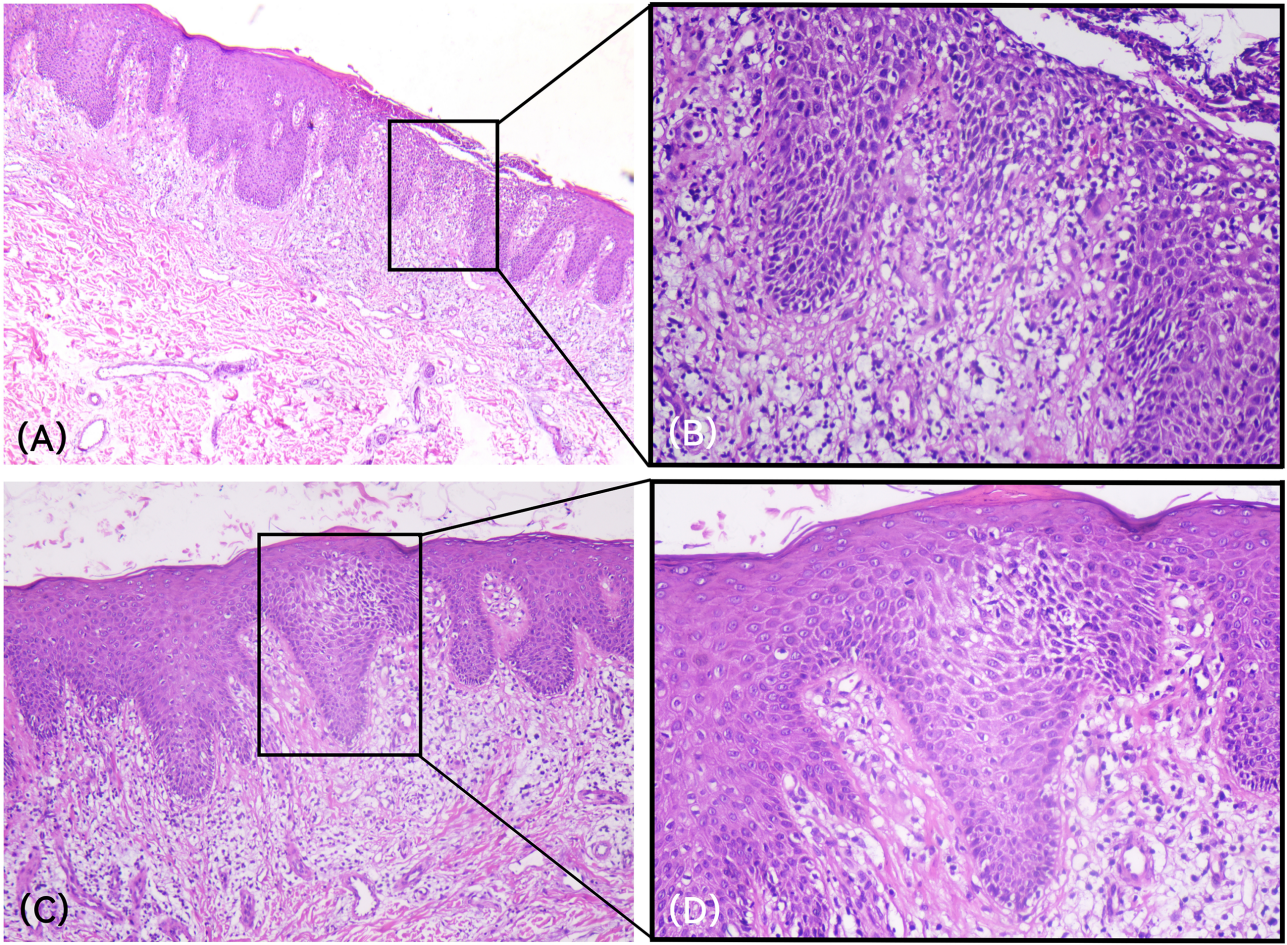
Histopathological findings. **(A, B)** Histopathological examination revealed hyperkeratosis with focal parakeratosis, psoriasiform epidermal hyperplasia, along with liquefactive degeneration of basal cells and lymphocytic infiltration in the superficial dermis (H&E; 2A: 40×, 2B: 200×). **(C, D)** Intercellular edema and spongiosis were observed in the epidermis (H&E; 2C: 100×, 2D: 200×).

Laboratory investigations revealed no significant abnormalities in complete blood count, liver and kidney function, or blood biochemistry. The serum total IgE level was elevated at 315.68 IU/mL. Two weeks after guselkumab injection, a skin biopsy from an erythematous lesion on the left upper arm. Histopathological examination showed hyperkeratosis with focal parakeratosis, accumulation of neutrophils in the superficial epidermis, psoriasiform hyperplasia, spongiosis, liquefaction degeneration of basal cells, and lymphocytic infiltration in the superficial dermis (**Figure 2**). Based on the clinical and histopathological findings, the diagnosis of paradoxical eczema induced by guselkumab was established.

**Figure 3:**
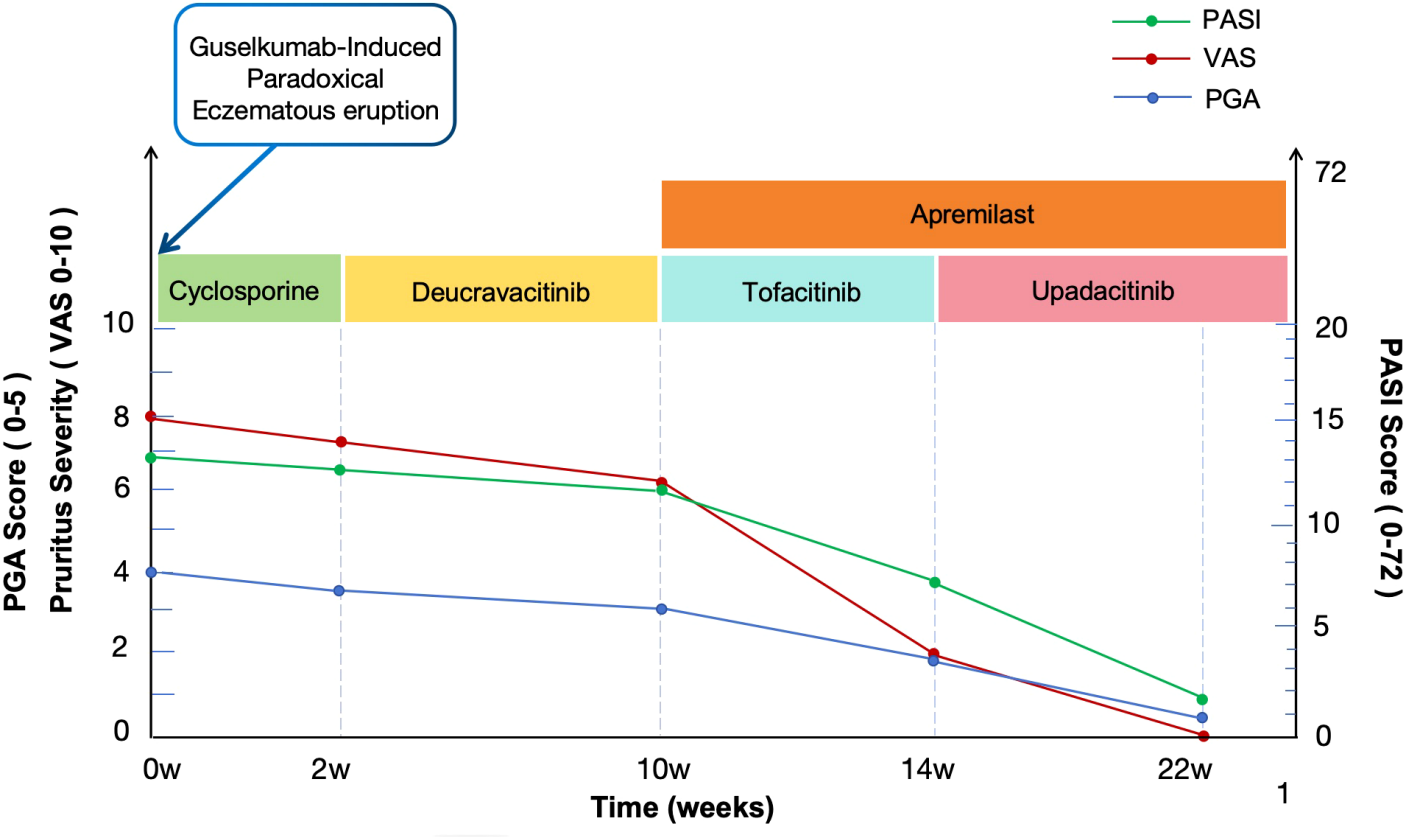
The patient’s clinical course during treatment.

Initially, cyclosporine (125 mg twice daily) was administered as a short-term intervention to rapidly control the inflammatory state. However, after two weeks of treatment, no significant improvement in skin lesions or pruritus was observed, with a PASI score of 12.1 (**Figures 1D–F**). Given the patient’s history of hypertension, the therapy was subsequently switched to the TYK2 inhibitor deucravacitinib (6 mg once daily) for eight weeks. Although the condition did not worsen, no significant further improvement was observed, PASI score 11.9 (**Figures 1G–I**). Given the previous partial response to tofacitinib, the patient was started on a combination of tofacitinib and apremilast (titrated to 30 mg twice daily). After four weeks, the patient achieved significant clinical improvement, with resolution of edema and erythematous plaques and substantial relief of pruritus, PASI score 7 (**Figures 1J–L**). However, considering the patient’s advanced age and long-term safety concerns, coupled with persistent and recurrent pruritic symptoms, the maintenance regimen was ultimately switched to upadacitinib (15 mg once daily) in combination with apremilast (**Figures 1M–O**). The patient’s clinical course during treatment is shown in **Figure 3**. Following 8 weeks of this combination therapy, his PASI score was 1.8 with stable condition and no new lesions. Pruritus was completely relieved (VAS score 0/10), and sleep quality returned to normal with no restrictions on daily activities. No adverse reactions were observed through week 12 of combination therapy. The patient’s blood pressure was well-controlled, with no abnormal changes in laboratory indicators, and the patient expressed high satisfaction with the treatment outcome.

## 3 Discussion

In recent years, with the increasing use of biologics in clinical practice, their associated adverse effects have drawn growing attention. A study reported that the incidence of paradoxical eczema in psoriasis patients receiving biologic therapy is approximately 1% (3). Specifically, the incidence rate associated with IL-23 inhibitors is only 0.56 per 100, 000 person-years, which is significantly lower than that of TNF-α inhibitors (0.94) and IL-17 inhibitors (1.22) (3). Guselkumab is a fully human IgG1λ monoclonal antibody that selectively targets the p19 subunit of IL-23 and exerts its therapeutic effects in psoriasis by modulating the IL-23/Th17 axis (4). The mechanism by which guselkumab induces paradoxical eczema has not been fully elucidated but may be related to its inhibitory effect on IL-23, which influences TNF-α and IFN-α signaling. IL-23 promotes the upregulation of TNF-α via Th17 cells, and TNF-α can inhibit the secretion of the proinflammatory cytokine IFN-α by plasmacytoid dendritic cells (pDCs) (2). By selectively inhibiting IL-23, guselkumab may attenuate the suppressive effect of TNF-α on IFN-α, leading to IFN-α overexpression. This subsequently activates pathological T cells and chemokines such as CXCL10 and CXCL9, thereby sustaining and amplifying local skin inflammation in paradoxical reactions. Furthermore, IL-23 inhibition may induce an paradoxical inflammatory reactions from the Th1/Th17 to Th2 phenotype, which may collectively contribute to the development of paradoxical eczema (5).

In this case, the patient developed significant pruritus three days after the first injection of guselkumab, which progressively worsened over the following two weeks, accompanied by eczematous lesions. Subsequent histopathological examination revealed spongiotic edema and liquefactive degeneration of basal cells, consistent with paradoxical eczema. Previously reported cases of guselkumab-induced paradoxical eczema had an onset ranging from 10 weeks to 2 years (6). To our knowledge, this case represents the shortest reported interval between guselkumab initiation and the development of paradoxical eczema. The rapid onset may be associated with the patient’s elevated total serum IgE level and a hypersensitive immune state, which indicated a subclinical Th2-skewed immune background. This rendered the patient highly susceptible to the immune microenvironmental perturbations upon IL-23 blockade, which precipitated rapid Th2 immune escape after Th1/Th17 pathway suppression, leading to the acute onset of paradoxical eczema (7). Furthermore, a differential diagnosis was still needed to distinguish with hypersensitivity reactions, eczematous psoriasis and contact dermatitis. The patient had no urticaria, angioedema or typical drug eruptions, nor systemic symptoms including fever and lymphadenopathy, without typical keratinocyte necrosis on histopathology, and along with elevated IgE suggesting Th2-skewed paradoxical eczema (5). what’s more, the patient had no prior history of psoriatic plaques transforming into eczematous lesions; distinct eczematous changes occurred only after biologic administration with a clear temporal correlation suggesting not aeczematized psoriasis. Finally, the symmetric lesions and no identifiable allergen exposure did not support a diagnosis of classic contact dermatitis. Therefore, the diagnosis of guselkumab-induced paradoxical eczema was confirmed. The current priority is to maintain control of the underlying disease while rapidly alleviating the paradoxical reaction. The treatment strategy was determined based on the following considerations. First, the patient declined further biologic therapy due to fear of exacerbating the paradoxical eczema, as switching to alternative biologics (such as IL-17A or IL-17A/F inhibitors) carries a potential risk of worsening the underlying Th2-driven inflammation. And the patient expressed a preference for oral medications. Second, the patient had previously shown a suboptimal response to conventional therapy with cyclosporine. Furthermore, JAK inhibitors have been reported in the literature to demonstrate efficacy in managing various paradoxical reactions induced by biologics (6). Therefore, a JAK inhibitor was ultimately chosen for ongoing management. We initiated treatment with deucravacitinib, a novel JAK inhibitor targeting tyrosine kinase 2 (TYK2). It not only maintains psoriasis stability by blocking the IL-23/Th17 axis—a core pathway in psoriasis pathogenesis—but also exerts potential therapeutic effects on various immune-mediated inflammatory dermatoses through modulation of signaling pathways involving type I interferons, IL-12, IL-23, IL-10, and IL-6 (8). Furthermore, its potential adverse effects on the cardiovascular system, liver, and kidneys are lower than those of conventional immunosuppressants such as cyclosporine. However, after 8 weeks of deucravacitinib treatment, the patient’s condition stabilized without further deterioration, but no significant clinical improvement was observed. The suboptimal response to TYK2 inhibition in this case may result from aberrant activation of specific immune pathways or failure to effectively block the complex inflammatory network. Although TYK2 inhibitors can block downstream IL-23/IL-12 signaling, their efficacy in controlling pre-existing Th2 polarization and IFN-γ-mediated inflammation is limited. Moreover, TYK2 inhibitors have high selectivity for TYK2 and exhibit weaker inhibition of other JAK family signaling pathways involved in eczema pathogenesis. While this outcome was not as expected, it provides valuable clinical guidance. In this specific patient, inhibition of TYK2-mediated signaling was insufficient to reverse established Th2-skewed paradoxical eczema, indicating the need for more direct and potent blockade of downstream Th2 effector cytokines.

In contrast, pan-JAK inhibitors such as tofacitinib or the highly selective JAK1 inhibitor upadacitinib offer broader blockade of pro-inflammatory cytokine signaling, including IL-4, IL-13, IL-31, IL-22, IFN-γ, IL-6, IL-7, and IL-17 (9). Tofacitinib (10 mg twice daily) has shown comparable efficacy to TNF-α inhibitors such as etanercept and even superior to conventional treatments like methotrexate in the treatment of psoriasis (10). Although not a first-line therapy for psoriasis, tofacitinib represents a viable therapeutic option for patients who have shown a suboptimal response to conventional systemic treatments and have developed paradoxical inflammatory reactions, following biologic therapy. After excluding conventional immunosuppressants with cardiovascular/renal risks and the patient’s refusal of biologics, we initiated off-label JAK inhibitor therapy with informed consent. We also optimized the treatment regimen by switching the pan-JAK inhibitor tofacitinib to the highly selective JAK1 inhibitor upadacitinib, which has lower off-target inhibition of JAK2/3. This reduces the risk of cardiovascular and thrombotic events, making it more suitable for elderly patients with cardiovascular comorbidities. By targeting JAK1, upadacitinib not only effectively blocks the Th2 inflammatory pathways involved in the pathogenesis of paradoxical eczema (IL-4, IL-13, IL-31), but also downregulates the expression of cytokines associated with the Th17/Th1 pathways in psoriasis, such as IL-22 and IFN-γ, thereby reducing keratinocyte hyperproliferation. This dual action alleviates paradoxical eczema while providing control of the psoriasis (11). Apremilast modulates inflammatory cytokine expression such as TNF-α by increasing intracellular cyclic adenosine monophosphate (cAMP) levels and activating the cAMP pathway, and has been increasingly used in recent years for the treatment of various inflammatory and immune-mediated skin diseases (12). Its favorable safety profile and lack of requirement for intensive laboratory monitoring make it particularly suitable for special populations and combination therapies. Although monotherapy with a JAK inhibitor was contemplated, apremilast was continued in light of the favorable clinical response and to mitigate the risk of psoriasis recurrence. The two agents act on different steps of the inflammatory cascade, enabling more comprehensive blockade of the complex inflammatory networks underlying paradoxical eczema and psoriasis, thereby overcoming the limitation of single-target therapies that that may inadequately cover multiple inflammatory pathways and lead to suboptimal efficacy. In this case, the combination therapy with upadacitinib and apremilast resulted in significant improvement of eczematous lesions and pruritus, along with significant improvement in psoriatic plaques, without any adverse events. The combination regimen was designed based on the patient’s prior treatment history and individual treatment preferences, retaining effective therapeutic components while minimizing medication-related risks, improving treatment adherence, and ensuring clinical efficacy.

As a single-case report, this study has inherent limitations. Potential confounding factors, including the patient’s advanced age and hypertension, may have influencedtreatment choices. Although we have excluded common underlying diseases that might affect cutaneous inflammatory status or trigger such adverse reactions, the possibility of occult underlying conditions cannot be completely ruled out. The findings require validation in large-scale clinical studies with long-term follow-up, incorporating standardized quality-of-life assessments and immune profiling.

## 4 Conclusion

For some patients with psoriasis, biologic or targeted monotherapy may be insufficient to achieve or maintain disease control. Although dose escalation can enhance efficacy, it may also increase the risk of adverse effects. In patients who develop biologic-induced paradoxical reactions, combining a JAK inhibitor with a PDE4 inhibitor may offer therapeutic benefits by targeting multiple inflammatory pathways. This strategy not only helps suppress psoriatic activity but also effectively counteracts paradoxical inflammatory reactions. The efficacy and potential synergistic effects of such combination therapy warrant further investigation in larger studies with monotherapy comparator arms.

## Data Availability

The original contributions presented in the study are included in the article/supplementary material. Further inquiries can be directed to the corresponding authors.

## References

[1] Munera-Campos M Ballesca F Carrascosa JM . Paradoxical reactions to biologic therapy in psoriasis: a review of the literature. Actas Dermosifiliogr Engl Ed. (2018) 109:791–800. doi: 10.1016/j.adengl.2018.09.012. PMID: 29903464

[2] Reyn B Hillary T Gils A . Eczematous eruption after guselkumab treatment for psoriasis. JAAD Case Rep. (2019) 5:973–5. doi: 10.1016/j.jdcr.2019.09.005. PMID: 31687468 PMC6820255

[3] Al-Janabi A Alabas OA Yiu ZZN Foulkes AC Eyre S Khan AR . Risk of paradoxical eczema in patients receiving biologics for psoriasis. JAMA Dermatol. (2024) 160:71–9. doi: 10.1001/jamadermatol.2023.4846. PMID: 38055239 PMC10701661

[4] Liakos W Toussi A Le ST Ma C Merleev A Marusina AI . Anti-interleukin 23-based therapies may shift cutaneous immune responses: a case of new-onset contact dermatitis to carbamates after guselkumab for psoriasis. Dermatitis. (2021) 32:e113–e5. doi: 10.1097/der.0000000000000765. PMID: 34138776

[5] Al-Janabi A Foulkes AC Mason K Smith CH Griffiths CEM Warren RB . Phenotypic switch to eczema in patients receiving biologics for plaque psoriasis: a systematic review. J Eur Acad Dermatol Venereol. (2020) 34:1440–8. doi: 10.1111/jdv.16246. PMID: 31997406

[6] Moreno-Davila H Gamez-Siller P Franco-Marquez R Garcia-Muniz JA Galarza-Delgado DA Cardenas-de la Garza JA . Eczematous reaction to guselkumab successfully treated with upadacitinib. J Eur Acad Dermatol Venereol. (2025) 40:499–501. doi: 10.1111/jdv.20857. PMID: 40668070

[7] Bavbek S Pagani M Alvarez-Cuesta E Castells M Dursun AB Hamadi S . Hypersensitivity reactions to biologicals: an EAACI position paper. Allergy. (2022) 77:39–54. doi: 10.22541/au.161718382.20133527/v1. PMID: 34157134

[8] Shang L Cao J Zhao S Zhang J He Y . TYK2 in immune responses and treatment of psoriasis. J Inflammation Res. (2022) 15:5373–85. doi: 10.2147/jir.s380686. PMID: 36147687 PMC9488612

[9] Mohamed MF Bhatnagar S Parmentier JM Nakasato P Wung P . Upadacitinib: mechanism of action, clinical, and translational science. Clin Transl Sci. (2024) 17:e13688. doi: 10.1111/cts.13688. PMID: 37984057 PMC10771099

[10] Berekmeri A Mahmood F Wittmann M Helliwell P . Tofacitinib for the treatment of psoriasis and psoriatic arthritis. Expert Rev Clin Immunol. (2018) 14:719–30. doi: 10.1080/1744666x.2018.1512404. PMID: 30118353

[11] Martinez-Molina M Lluch-Galcerá JJ Carrascosa JM . Response to upadacitinib in a patient with palmoplantar psoriasis. Eur J Dermatol. (2023) 33:301–2. doi: 10.1684/ejd.2023.4489. PMID: 37594341

[12] Carmona-Rocha E Rusinol L Puig L . Exploring the therapeutic landscape: a narrative review on topical and oral phosphodiesterase-4 inhibitors in dermatology. Pharmaceutics. (2025) 17:91. doi: 10.20944/preprints202412.1339.v1. PMID: 39861739 PMC11769339

